# Dietary Restrictions and Cancer Prevention: State of the Art

**DOI:** 10.3390/nu17030503

**Published:** 2025-01-29

**Authors:** Greta Caprara, Rani Pallavi, Shalini Sanyal, Pier Giuseppe Pelicci

**Affiliations:** 1 Department of Experimental Oncology, European Institute of Oncology (IEO), Istituto di Ricovero e Cura a Carattere Scientifico (IRCCS), 20139 Milan, Italy; 2 Brien Holden Eye Research Centre, L. V. Prasad Eye Institute, Hyderabad 500034, India; 3 The Operation Eyesight Universal Institute for Eye Cancer, L. V. Prasad Eye Institute, Hyderabad 500034, India; rani.pallavi@lvpei.org (R.P.); sanyal.shalini@gmail.com (S.S.)

**Keywords:** dietary restriction, calorie restriction, fasting, cancer, prevention, diet, nutrition, overweight, microbiota

## Abstract

Worldwide, almost 10 million cancer deaths occurred in 2022, a number that is expected to rise to 16.3 million by 2040. Primary prevention has long been acknowledged as a crucial approach to reducing cancer incidence. In fact, between 30 and 50 percent of all tumors are known to be preventable by eating a healthy diet, staying active, avoiding alcohol, smoking, and being overweight. Accordingly, many international organizations have created tumor prevention guidelines, which underlie the importance of following a diet that emphasizes eating plant-based foods while minimizing the consumption of red/processed meat, sugars, processed foods, and alcohol. However, further research is needed to define the relationship between the effect of specific diets or nutritional components on cancer prevention. Interestingly, reductions in food intake and dietetic restrictions can extend the lifespan of yeast, nematodes, flies, and rodents. Despite controversial results in humans, those approaches have the potential to ameliorate health via direct and indirect effects on specific signaling pathways involved in cancer onset. Here, we describe the latest knowledge on the cancer-preventive potential of dietary restrictions and the biochemical processes involved. Molecular, preclinical, and clinical studies evaluating the effects of different fasting strategies will also be reviewed.

## 1. Introduction

Globally, an estimated 20 million new cancer cases and almost 10 million cancer deaths occurred in 2022. These numbers are projected to increase by approximately 60% over the next two decades, with the global burden expected to exceed 35 million new cancer cases and approximately 18.5 million cancer deaths by 2050 (with the greatest increases happening in low- and middle-income countries) [1]. Hence, the continuous growth and aging of the world’s population, along with advances in early detection and treatment, made cancer survivors a significant part of the current population: in 2022, more than 53 million people worldwide were estimated to have lived 5 years after a cancer diagnosis [1]; considering the expected global increase in cancer burden, it is reasonable to guess this number will also increase by 2050.

Primary prevention has long been identified as a key control strategy to reduce the global cancer burden. In fact, as documented by several studies, dietary components and lifestyle behaviors are crucial modifiable factors in the modulation of cancer risk. In fact, there is growing evidence that 30–50% of all cancers can be prevented by avoiding unhealthy diets, physical inactivity, alcohol consumption, tobacco use and obesity. Particularly, overweight and obesity are causally associated with the development of at least 13 different types of cancer [2,3,4].

Beyond the impact of body fatness, the combination of specific dietary components, as well as physical activity, is known to be able to affect one’s susceptibility to cancer development [2,5]. In agreement with that, many international organizations have established cancer prevention guidelines that highlight the importance of following an overall “healthy dietary pattern” based on the regular consumption of plant-based foods (whole grains, legumes, vegetables, fruits, etc.), a moderate amount of fish, dairy, and poultry, and a low consumption of red and processed meat, salt-preserved foods, sugars, alcohol, and pastry [2]. Even though it is still insufficient to make specific recommendations, increasing evidence seems to indicate that following the same advice could be appropriate even after a cancer diagnosis [2,6,7,8,9]. Proper diets and healthy lifestyles, in fact, are associated with better health-related quality of life in cancer survivors [10,11]. Further studies, however, are required to better clarify the existing relationship between the effect of specific lifestyle interventions and dietary components on cancer prevention, relapse and outcome [11].

A reduction in food intake and/or dietetic restrictions, without malnutrition, have been reported to exert positive effects on health and lifespan. This has been undoubtedly demonstrated, for instance, in yeast, nematodes, flies, and rodents, where Calorie Restriction (CR) results in life extension. However, controversial results have been observed in primates and, particularly, in humans [12,13,14].

A growing body of evidence has shown that CR triggers similar biological pathways and metabolic adaptations as fasting, where the term “fasting” encompasses many different types of regimens, such as: Full Fasting (FF), Alternate-Day Fasting (ADF), Alternate-Day Modified Fasting (ADMF), Periodic Fasting (PF), Fasting-Mimicking Diets (FMDs), Intermittent Fasting (IF), and Time-Restricted Feeding (TRF) [15].

Specifically, both fasting and CR might exert broad direct and indirect systemic effects on peculiar growth factor signaling pathways, energy homeostasis, redox state and autophagy modulation, and hormone and cytokines levels, thus affecting the onset of age-related diseases such as cancer [16,17].

Nevertheless, there is much to be learned before being able to define evidence-based and personalized nutritional recommendations to prevent cancer development and recurrence. In the following paragraphs of this literature review, we will focus our attention on the latest emerging knowledge and perspective about the effects of CR and fasting on cancer prevention. Cellular, molecular, preclinical and clinical studies will be reviewed, described and discussed, accordingly.

## 2. Nutrition and Cancer

### 2.1. Diet and Cancer: Epidemiological Evidence

Cancer is a genetic disease caused by alterations to genes controlling cells’ growth and proliferation. Cancer-related genetic mutations can be inherited, due to accidental mistakes occurring during cell division, or arise from particular endogenous (by-products of metabolism, free radicals, etc.) or exogenous exposures [18,19]. The latter are mostly represented by radiations (X-rays, gamma rays, UV radiations, etc.), chemicals (tobacco smoke, aflatoxins, arsenic, asbestos, etc.), and infectious agents (HPVs, EBV, Helicobacter pylori, etc.). However, a large and growing body of literature has demonstrated that alcohol consumption, physical inactivity, overweight, obesity, and unhealthy diets are other kinds of exogenous exposures able to increase the risk of cancer [2,20,21,22,23]. In agreement with that, more than 50% of all cancer cases are attributable to the abovementioned behavioral risk factors [24].

Despite in vitro and in vivo research having shown that some isolated nutrients, specific substances, and/or molecules may either exert a pro- or anti-tumor activity, epidemiologic studies have not ultimately indicated that any single dietary component, per se, is able to cause or protect against cancer development in humans [2]. Nonetheless, evidence derived from several epidemiological studies, such as one of the largest cohort studies in the world, namely, the European Prospective Investigation into Cancer and Nutrition (EPIC) study [25], and the latest scientific research available derived from the Third Expert Report from the World Cancer Research Fund and the American Institute of Cancer Research (WCRF/AICR) [2], showed that the combination of specific dietary components, meaning the overall diet composition, is able to affect the susceptibility to cancer development. Thus, it is now widely accepted by the scientific community that a dietary pattern mainly following the traditional Mediterranean nutritional model [26,27], which is based on the regular consumption of plant-based foods (whole grains, legumes, vegetables, fruits, etc.), a moderate amount of fish, dairy, eggs, and poultry, and a low consumption of red and processed meat, salt-preserved foods, sugars, alcohol, and pastry, can decrease the risk of tumor development [28,29]. Indeed, people who follow diets that emphasize the consumption of plant-based foods tend to have a lower risk of cancers such as breast [30], colorectal [31], and prostate [32]. This is likely due to the fact that these types of regimes are particularly high in fiber-rich foods, which promote gut health and have anti-inflammatory and antioxidant properties that may reduce the risk of cancer [33,34], whereas red meat and diets rich in sugary foods, refined carbohydrates, high-fat diets and high calorie energy dense foods increased the risk of tumors [35].

In agreement with that, the WCRF Expert Panel, which conducted, for many years, the most rigorous analyses of the published literature linking cancer risk to diet, nutrition and physical activity, provided ten authoritative evidence-based cancer prevention recommendations aimed at decreasing the global burden of cancer [36]. Those essentially, advise keeping the weight within a healthy range and avoiding weight gain in adulthood; being physically active; reducing the consumption of energy-dense foods (“fast foods” and other processed foods high in fat, starches, or sugars), red and processed meat, sugar-sweetened drinks, and alcohol; and making healthy foods (such as wholegrains, vegetables, fruit, and beans) a major part of the habitual diet [36].

Although further studies are required to understand how specific dietary and lifestyle patterns can affect cancer survivorship, papers have shown that a healthy diet and lifestyle are associated with better quality of life even after a cancer diagnosis [10,11]. Therefore, despite insufficient data to make specific nutritional and lifestyle recommendations for patients and cancer survivors, many dedicated scientific organizations have established that following the same general nutritional advice given for cancer prevention could be appropriate [2,6,7,8,9].

### 2.2. Effect of Overweight on Tumor Risk

The proportion of adults affected by overweight and obesity is increasing both in high-income and low-/middle-income countries, where it is now on the rise, mostly in urban settings. According to the World Health Organization (WHO) (2016), more than 1.9 billion adults (18 years and older) are overweight (defined as a body mass index—BMI—between 25 and 29.9), of which 650 million are affected by obesity (defined as a BMI of 30 or above). That corresponds to 39% of overweight adults and 13% living with obesity [37]. Moreover, the WHO estimated that 38.2 million children, under the age of 5 years, were overweight or obese in 2020, while over 340 million children and adolescents, aged 5–19, were overweight or obese, in 2016 [37]. Globally, the prevalence of “overweight” and “obesity” and the number of affected individuals will continue rising during the next decade, thus rendering those subjects more prone to developing obesity-related non-communicable diseases such as cardiovascular pathologies, type 2 diabetes, and cancer [37,38,39,40,41,42].

Especially, being overweight, or living with obesity throughout adulthood, is causally linked to the development of many cancers, namely, mouth, pharynx, and larynx cancer, esophageal, stomach, pancreatic, gallbladder, liver, colorectal, breast (post-menopause), ovarian, endometrial, prostate, and kidney cancer [39,42,43].

The biological mechanisms and molecular metabolic pathways underlying the relationship between overweight/obesity and malignant transformation are complex and multifactorial and still demand further investigation. Additionally, it appears that specific key molecular players are involved in the initiation of distinct cancer entities and genders, thus preventing the designation of any common determinants of carcinogenesis. Even though several mechanisms and players have been suggested to be involved in obesity-associated cancer development, a general mechanism that can explain the interconnection of obesity and cancer is still far from being achieved; therefore, researchers hypothesized that it is possibly a combination of physiological and homeostatic alterations, occurring during obesity, that collectively increases cancer risk. Those changes mainly include abnormalities affecting the inter-tissue crosstalk and generating a tumor-promoting milieu. Accordingly, several hormones, metabolites, growth factors, signaling pathways, and immune and inflammatory mediators are dysregulated and/or display abnormal expression levels [44,45].

Some of the key mechanisms and players possibly involved in obesity-associated cancers are described below.

1. Adipokines

Nowadays, Adipose Tissue (AT) is considered an endocrine organ able to secrete several enzymes and mediators, thus regulating energy homeostasis at many levels. During overweight and obesity, AT becomes dysfunctional due to the overload of adipocytes with lipids and the massive invasion of immune cells, manifesting an altered production of the bioactive molecules adipokines. Briefly, AT starts to release high levels of cell-signaling proteins (leptin, resistin, and pro-inflammatory cytokines) able to activate pathways involved in cell proliferation, survival, migration, and angiogenesis (such as NF-κβ, JAK/STAT, MAPK, PI3K/Akt, VEGF, and Wnt/β-catenin) while inhibiting apoptosis. Furthermore, circulating levels of adiponectin (APN), an adipokine with anti-inflammatory, antiproliferative and proapoptotic properties, are dramatically reduced in obesity. Altogether, those metabolic alterations contribute to creating a microenvironment able to promote tumor development and progression [46].

2. Pro-inflammation conditions and immune system

Obese AT is also characterized by a massive infiltration of cells of the innate immune system, particularly, the so-called “pro-inflammatory M1 macrophages”. The dysfunctional adipocytes, releasing free fatty acids and inflammatory mediators, promote the polarization of “anti-inflammatory M2 macrophages” to a pro-inflammatory condition. In this setting, both adipocytes and immune cells enhance the secretion of pro-inflammatory cytokines like IL-1β, IL-6 and TNF-α, thus exacerbating the inflammatory state, which, in turn, can stimulate other pro-inflammatory signaling pathways, angiogenesis, and tissue remodeling. Mounting evidence supports the hypothesis that chronic inflammation exhibits a pro-tumorigenic potential by stimulating cell proliferation, survival, tumor development, and/or progression [47,48].

Besides that, obesity has been shown to also affect the immune response against cancer. Indeed, recent studies showed that overweight and obesity conditions are associated with dysfunction of the innate and adaptive immune systems, negatively affecting the antitumor and cytotoxic functions of natural killer (NK), CD8+T, and dendritic cells (DCs) [49]. Since the immune system exerts a pivotal activity in cancer surveillance, the obesity-related alterations in the immune response may further contribute to the risk of developing cancer in overweight subjects [50].

3. Hormones

AT is also a source of hormone production; for instance, circulating levels of estrogens directly correlate with the amount of body fat, and their overproduction has been linked to an increased risk of developing endometrial, ovarian, and post-menopausal breast cancers. The enzyme aromatase, which converts androgens to estrogens, is found in peripheral AP, and its expression is further induced by excess pro-inflammatory cytokines, typically produced by obese adipose tissue, thereby promoting even more hyper-estrogenemia. Estrogens could stimulate tumor progression in many ways: inhibiting apoptosis, fueling mitogenesis or inducing genomic instability [51].

4. Insulin and Insulin-like Growth Factor-1 (IGF-1) signaling

A common consequence of an increased BMI is hyperinsulinemia, which also stimulates the expression of Insulin-like Growth Factor-1 (IGF-1). Insulin and IGF-1, respectively, bind to the insulin receptor (IR) and IGF-1 receptor (IGF1R), whose major downstream effectors are the PI3K/AKT and MAPK signaling pathways [52]. Interestingly, the PI3K/AKT/MAPK cascade is one of the signal mediators of leptin, adiponectin, and many inflammatory cytokines, and is also a pathway very frequently mutated in human cancers. Indeed, disruption of the PI3K/AKT/MAPK cascade has been shown to suppress tumor activity [53,54,55].

5. Gut microbiota

Mounting evidence has established that diet composition can significantly impact microbiota structure and regulate, in turn, host physiological responses and health conditions. Briefly, differences in the gut microbial phylae have been observed between healthy and obese subjects: Firmicutes and Bacteroidetes are often most abundant under healthy-weight conditions, while Enterobacteriaceae were found to be increased in overweight/obese subjects [56]. The latter is known to release large amounts of lipopolysaccharide (LPS), thus stimulating a pro-inflammatory milieu, which might exacerbate the chronic inflammatory state characterizing the obese conditions. Moreover, an altered microbiota can contribute to increasing the risk of developing certain cancers by provoking barrier failure and/or trigger accumulation of toxic compounds [57].

In agreement with the abovementioned biological mechanisms, studies have shown that weight loss, particularly if greater than 10%, can reverse the obesity pro-inflammatory state by reducing, for instance, the levels of TNF-α, IL-6, and the leptin-to-adiponectin ratio. Even though further research is still needed, mounting evidence suggests that in people with a higher BMI, weight loss is helpful in reducing cancer risk [40,58,59,60]. Moreover, successful bariatric surgery, attaining significant and long-term weight loss, is effective in decreasing the incidence of obesity-associated cancer [61,62,63]. Interestingly, as we will see in more detail in the following paragraphs, CR involving a 30% reduction in the daily caloric intake and CR without malnutrition have been shown to prevent cancer in preclinical studies [64].

It has been suggested that adipose tissue and its microenvironment may stimulate not only carcinogenesis, but also tumor progression and metastasis development. Moreover, overweight and obesity may also lead to a poorer treatment response, worsened prognosis, and increased cancer-related mortality [65,66]. For instance, there is evidence to indicate that elevated body fatness predicts a poor outcome in breast cancer (BC) survivors [6,7,67]. The exact cause of this association is still unclear; it has been hypothesized that the impact of overweight and obesity can increase the risk of developing other chronic diseases, which may reduce the overall survival; moreover, obesity-associated chronic inflammation may also enhance tumor progression. Despite that, it is not still possible to conclude with certainty that body fat reduction would be able to improve prognosis in BC survivors [6,7]. In addition, there is limited evidence that obesity may be associated with a poor prognosis in other cancers. In fact, links have been found between being overweight at diagnosis and longer survival in patients with certain types of tumors, including melanoma and lung. The association between higher BMI and improved survival outcome is still unexplained and needs to be addressed in future studies [6,66,68,69]. In conclusion, whether and to what extent cancer patients with a higher BMI could benefit from weight loss strategies is unclear; so, no recommendations have been made yet in this regard. Nevertheless, the Expert Panel of the WCRF came to the conclusion that, unless otherwise advised by a health professional, cancer survivors who have completed treatments are encouraged to maintain a healthy weight [6].

An increasing amount of evidence is showing that a higher BMI is causally related to an enhanced risk of cancer; accordingly, weight loss is tied to a drop in cancer risk. But what about lean people? Is it possible that a healthy diet, which allows obese people to lose and maintain a normal weight, may also be useful for slim subjects, remodeling, through some specific dietary components, the pathways implicated in the development and progression of cancer? Moreover, could fasting regimens, which regulate the activity of specific metabolic pathways responsible for nutrient sensing and cellular stress responses, also be beneficial for healthy-weight people to prevent malignant transformation?

### 2.3. Nutrient-Sensing Mechanisms: Cellular Processes and Molecular Pathways Involved

Nutrient-sensing pathways are crucial for governing cellular metabolism, growth, and survival in accordance with fluctuations in nutrient availability. When these pathways are disrupted, they are associated with the onset of cancer. Consequently, it comes as no surprise that individuals afflicted with metabolic disorders like diabetes, obesity, and hypertension, where one or more nutrient-sensing pathways are disrupted, consistently demonstrate an increased susceptibility to cancer development [70,71,72]. In this section, we will be exploring the intricate relationship between nutrient-sensing pathways and the risk of cancer. The primary emphasis will be on exploring how dietary patterns, particularly those characterized by increased intake of lipid, amino acid, and glucose-rich foods, can impact these pathways and their association with cancer risk.

#### 2.3.1. Insulin/Insulin-like Growth Factor 1 (IGF-1) Pathway and Cancer Risk

Sugar-rich food stimulates the Insulin/IGF1 signaling. The peptide hormones Insulin and IGF-1, by binding to their respective receptors (IR and IGF1R), initiate a cascade of intracellular signaling events through the PI3-kinase/Akt and the Ras/MAP kinase pathways. The activation of the Insulin/IGF1 pathway promotes glucose uptake, protein synthesis, and cell proliferation. Many population studies have indicated the correlation between the level of insulin and IGF1 and the risk of certain cancers. An observational study conducted in non-diabetic postmenopausal women found that increased insulin levels were a risk factor for developing BC regardless of estrogen levels [73]. Although there was no linear relationship between the level of IGF1 and the risk of BC in these patients, a possible curvilinear relationship was suggested. It seems that with the elevated level of insulin, the risk of post-menopausal BC increases up to 2-fold [74]. A meta-analysis of epidemiological studies also confirmed the increased risk of breast cancer associated with elevated insulin levels. In addition, this analysis also revealed the increased risk of prostate cancer [75]. More recently, a study involving nearly 400,000 participants confirmed an association between elevated blood IGF-1 levels and an increased risk of multiple cancer types, further emphasizing the link between IGF-1 levels and cancer risk [76].

#### 2.3.2. Mechanistic Target of Rapamycin (mTOR) Pathway and Cancer Risk

Given the role of the mTOR pathway in maintaining dormancy, a characteristic of both quiescent stem cells and quiescent cancer stem cells (CSCs), it plays a central role in the emergence of latent cancer cells [77]. Consequently, it is unsurprising that mutations in components of the mTOR pathway are frequently observed in cancer [78]. Additionally, evidence suggests an association between mTOR polymorphisms and a predisposition to various cancers. Specific mTOR SNPs, such as rs1883965, rs1034528, and rs17036508, are linked to lower mTOR transcript levels and an increased risk of cancer [79]. Recently, a large-scale study analyzed 28,847 single-nucleotide polymorphisms (SNPs) across 61 genes in the mTOR pathway for their impact on breast cancer risk. The study identified an association with intronic SNPs TSC2 rs181088346 and BRAF rs114729114 (OR = 1.53, 95% CI = 1.24–1.91) for overall breast cancer. Additionally, intronic SNPs PGF rs11542848 and MAPK3 rs78564187 were linked to an increased risk of Estrogen Receptor (ER)-negative breast cancer [80].

#### 2.3.3. AMP-Activated Protein Kinase (AMPK) Pathway and Cancer Risk

The AMPK pathway is central to cellular energy regulation and acts as a crucial nutrient sensor, balancing energy availability with metabolic needs. This pathway’s ability to suppress tumorigenic processes makes it essential for cancer prevention. However, disruptions in AMPK signaling—whether through mutations, downregulation, or dysregulated activation—can compromise its tumor-suppressive roles, thereby elevating cancer risk [81].

The regulation of mitochondrial respiration, nutrition transport, autophagy, differentiation, lifespan, and cell polarity are all significantly influenced by AMPK. Furthermore, because cancer cells are physiologically suited to survive, especially in the face of nutritional or energy deprivation, AMPK activation is an essential mechanism that promotes tumor cell survival. By blocking anabolic programs and mTORC1 signaling, AMPK activation facilitates the switch from anabolic to catabolic metabolism, promoting cell survival and development within tumors that experience the depletion of catabolic substrates [82].

When there is an excess of nutrients, pathways that support cellular growth and proliferation are over/hyperactivated, such as mTORC1 hyperactivation, which has been observed in a high percentage of human cancers. Chronic nutrient abundance keeps mTOR constantly active, promoting cell growth and reducing autophagy, a process critical for cellular cleanup. Without autophagy, damaged organelles and misfolded proteins can accumulate within cells, increasing the risk of genomic instability and mutations. This persistent anabolic environment contributes to an increased cancer risk as it supports unchecked cell proliferation while reducing cellular resilience against DNA damage and stress [83].

On the other hand, for yeast, normal mammalian cells, and mammals to survive famine, acute autophagy induction is essential. Although autophagy was first believed to be a tumor suppression mechanism, there are tumor types or subtypes—like hepatomas—where autophagy genes may be altered and their loss of function may encourage malignancy. These tumor types or subtypes have not yet been adequately described at the genomic level. The lack of autophagy results in oxidative stress, DNA damage response activation, and genomic instability, all of which are known to contribute to the development and spread of cancer [84].

### 2.4. Dietary Restriction Interventions and Cancer Incidence

Dietary restriction (DR) has long been considered a very robust means to extend healthspan and lifespan; it encompasses many kinds of dietary interventions that differently restrict the intake of energy and/or specific nutritional components [85]. DR interventions such as Calorie Restriction (CR), Fasting-Mimicking Diet (FMD) and Intermittent Fasting (IF) are studied in relation to cancer incidence due to their ability to influence biological pathways that are either involved or considered a risk factor for cancer. Both specific dietary components and total calorie intake have been linked to cancer incidence. The importance of dietary patterns/components in influencing cancer incidence dates back to the 1980s [86], with earlier research indicating that eating habits can substantially impact cancer risk, primarily through mechanisms involving inflammation, oxidative stress, and hormonal imbalance [87].

#### 2.4.1. Calorie Restriction (CR)

CR is the most studied intervention known to be able to extend lifespan and delay the onset of chronic and age-associated diseases, from yeast to mammals [88]. Specifically, CR refers to the reduction in total calorie intake (by 20 to 40%), without malnutrition (Table 1). The first study on CR was published in 1935, by McCay et al. [89], who demonstrated this intervention was able to nearly double rats’ lifespan by reducing the incidence of many chronic diseases, including tumors [89,90]. Other evidence of the protective effect of CR on cancer incidence in animals includes its impact on both spontaneously developing and chemically induced cancers [91,92]. Research on CR expanded rapidly over the 20th century [93,94], showing overall that this kind of DR enhances median and maximal lifespan and inhibits the occurrence of many spontaneous cancers—including liver cancer [95], skin cancers [96], lymphomas [97], and mammary tumors [98,99,100]—not only in rodents, but also in a variety of other lower species such as yeast, flies, worms, and fish [85,97,101].

Due to those promising findings, starting in the 1990s, studies on CR moved on to monkeys and humans.

However, despite its ability to decrease the incidence of many chronic diseases [12] and achieve a 50% reduction in the incidence of gastrointestinal adenocarcinoma in non-human primates [102], CR has also produced conflicting results in extending the life span of monkeys, and long-term CR has even shown adverse effects on human health, potentially increasing the risk of disease-related premature death [85,93,102,103,104,105].

Considering the potential adverse effects of CR on human health, researchers also studied other different dietary interventions (from those restricting calories almost completely, to those only limiting the availability of specific nutrients), with the potential to prevent cancer and other chronic diseases, while improving feasibility and long-term safe use (Table 1) [106,107].

Indeed, some of them appeared to be less invasive than CR and possibly more feasible and effective in promoting human health and lifespan, namely, FMD and IF (Table 1) [85,108,109].

#### 2.4.2. Fasting-Mimicking Diet (FMD)

FMD usually refers to a plant-based diet characterized by a very low intake of calories (between 300 and 1000 kcal/day), a low intake of proteins (11–14%) and sugars, and a relatively high intake of unsaturated fats. It has been designed to mimic the metabolic effects induced by water-only fasting while decreasing its nutritional risks [110,111,112,113,114]. FMD typically lasts 3–7 days; an ad libitum diet is fed between those periods. It can be followed a few times to 24 times a year, depending on the subjects’ characteristics. (Table 1) [110,111,112,113,114].

It is hypothesized that a FMD may promote cancer prevention in mouse models by reducing levels of IGF-1, insulin, leptin, glucose, visceral fat, and other disease and aging markers [13]. However, there are no direct studies on FMD’s effects specifically for cancer prevention in animal models or humans. Most existing studies demonstrate FMD’s ability to delay tumor growth or enhance the effects of chemotherapy and radiotherapy in cancer treatment [115,116]. Therefore, research focused on understanding the impact of FMD on cancer risk and prevention is warranted.

#### 2.4.3. Intermittent Fasting (IF)

IF alternates between periods of eating and fasting, which can last from 12–16 to 24 h. Many examples of IF exist, including (i) Alternate Day Fasting (ADF), which alternates a complete fasting day with a feast day; (ii) Modified Alternate Day Fasting (MADF), which restricts 75–85% of the total energy every other day; (iii) the 5:2 diet, which alternates 2 days per week of very low calorie restriction (500–700 kcal) with a 5-day ad libitum re-feeding period; and (iv) Time-Restricted Feeding (TRF), during which food consumption is restricted to 6–12 h per day every day (Table 1) [117,118,119,120,121,122].

In contrast to CR studies, IF studies in animal models have produced inconsistent results, possibly due to variations in the protocols used. An IF protocol involving two fasting days per week had no effect on the incidence of mammary tumor formation in DBA mice [123]. However, in cancer-prone p53 mice, a protocol with one fasting day per week significantly delayed tumor onset and reduced tumor metastasis [124]. Similarly, an IF protocol involving six cycles of one day of fasting followed by one day of feeding was able to reduce the development rate of both B-cell and T-cell acute lymphoblastic leukemia but showed no effect in a mouse model of acute myeloid leukemia [125]. A delayed onset of lymphoma was also observed with ADF in OF1 mice [126]. However, in a chemically induced rat model of hepatocellular carcinoma, IF protocols involving fasting periods followed by refeeding consistently resulted in higher incidences of liver cancer [127,128]. This effect was independent of the number of fasting and refeeding days or the cycles used. For example, protocols with five cycles of four days of fasting followed by ten days of refeeding [128], three cycles of three consecutive fasting days with eleven days of refeeding [128], or four days of fasting followed by refeeding [129], all showed an increase in liver cancer incidence. This may be attributed to a recent finding suggesting that increased stem cell regeneration and tumorgenicity during the refeeding phase could potentially raise cancer risk [130]. Further research is required to validate these findings across different cell and cancer types. Although few studies have examined the effect of TRF on cancer incidence, one study using the transgenic MMTV-polyoma middle T antigen model of mammary tumorigenesis showed that TRF, initiated at 8 weeks, inhibited mammary tumor initiation [131,132].

In Section 3, we will report in more detail on the molecular background and preclinical, clinical, and epidemiological studies describing the effects of CR, FMD, and IF in cancer prevention.

**Table 1:** Dietary restriction approaches studied over the past years for their potential in ameliorating health- and lifespan, and preventing cancer and other chronic diseases.

Type of Dietary Restriction			Schedule	Description
Calorie Restriction (CR)			Months–years.	Reduction in energy intake below the total amount of calories (20–40% below average) that would be needed to maintain a person’s current body weight without undergoing malnutrition [133,134,135].
Fasting/Water-only fasting			12 h weeks, very low or no calorie intake.	Fasting refers to a voluntary abstinence from some or all foods or foods and beverages for preventive, therapeutic, religious, spiritual, political, cultural, ethical or other reasons. Most fasting approaches allow limitless access to water; thus, they are called “Water-only fasting” (only water is consumed for a certain period of time) [133,135].
Prolonged Fasting (PF)			2–5 days of water-only fasting/7 days eating period (or longer).	Prolonged Fasting (PF), also called Long-Term Fasting (LTF), refers to fasting regimens lasting ≥4 consecutive days. Water-only fasting, followed by ad libitum re-feeding period [15,133,136,137,138,139].
Intermittent Fasting (IF)		Alternate Day Fasting (ADF)	24 h fast/24 h eating period.	It refers to alternating a day of eating ad libitum and a day of water-only fasting [133,134,136,140].
		Modified Alternate Day Fasting (MADF)	15–25% of total daily energy expenditure during 2 non-consecutive fasting days a week. Ad libitum food intake for the other 5 days.	It refers to alternating a day of eating ad libitum and a day of modified fasting. The term modified fasting refers to limiting energy intake to typically up to 25% of energy needs on modified fasting days. This form of fasting is similar to ADF, but with a 75–85% energy restriction, every other day. Ad libitum diet is followed on the other 5 non-fasting days [120,133,134,141].
		5:2 diet	2 days fast, or very low-calorie consumption (500–700 kcal) per week/5 days eating period.	Alternation of 2 days per week of very low-calorie consumption (500–700 kcal) with a 5-day ad libitum re-feeding period [134,136,142].
		Time-Restricted Feeding (TRF) or Time-Restricted Eating (TRE)	12–18 h fast/6–12 h eating period.	Food intake is restricted to 6–12 h per day, done daily. There is no explicit limit on energy intake during eating hours [15,117,133,134,136,143,144,145].
Fasting-Mimicking Diet (FMD)			3–7 continuous days of calorie-restricted diet/25-day eating period.	Any diet specifically composed to induce the metabolic effects of fasting while allowing for a potentially higher caloric intake, including solid foods. It usually refers to a plant-based, calorie-restricted diet with a maximum of approximately 1000 kcal/day that lasts 3–7 days. FMD is usually relatively low in refined sugars and starch, low in protein, and high in plant-based fats. This nutritional program is separated by 25 days of an ad libitum diet, and it is normally followed once or twice in a month [15,110,133].
Nutrient Restriction (NR)	Glucose ^#^ and Carbohydrate * Restriction		/	Carbohydrate consumption is restricted, relative to the average diet, and is replaced by food containing a higher percentage of fat and protein [134].
	Protein Restriction (PR)		/	Reduction in dietary protein intake without changing the average caloric intake [134].
	Amino Acid Restriction (AAR)		/	Specific restriction of amino acids that commonly include threonine, histidine, lysine, methionine, and branched-chain amino acids (BCAAs) [134].

^#^ Glucose restriction refers to a specific restriction of glucose intake instead of other forms of complex carbohydrates. * Very-low-carbohydrate diet (<10%), low-carbohydrate diet (<26%), moderate-carbohydrate diet (<26–44%) [146].

## 3. Calorie Restriction (CR), Fasting-Mimicking Diet (FMD) and Intermittent Fasting (IF) as Approaches to Prevent Cancer

### 3.1. Proposed Mechanisms and Evidence for the Anti-Tumor Activity of Calorie Restriction (CR)

The mechanisms underlying the anti-cancer benefits of CR involve multiple nutrient-sensing pathways, stress response systems, and metabolic adaptations, all of which play critical roles in modulating cellular proliferation and survival.

#### 3.1.1. CR: Cellular and Molecular Effects on Nutrient-Sensing Pathways

One of the fundamental effects of CR is its ability to modulate nutrient-sensing pathways, which are sensitive to changes in energy availability and orchestrate a range of cellular processes to adapt to nutrient scarcity. By influencing these pathways, CR can suppress tumorigenesis and promote cellular resilience against oncogenic stressors.

##### Fatty Acid Metabolism

According to reports, CR regimens show decreased expression of genes for fatty acid (FA) production and increased expression of genes for FA oxidation [147]. However, the metabolic signals and adaptations at play are not completely understood yet. CR has profound effects on various metabolic pathways, including lipid metabolism, which plays a critical role in cancer development and progression. By altering the regulation of fatty acids and lipids, CR influences tumorigenic processes such as cell proliferation, inflammation, and oxidative stress. The key nutrient-sensing pathways involved in fatty acid and lipid metabolism under CR conditions include AMP-activated protein kinase (AMPK), sirtuins, and peroxisome proliferator-activated receptors (PPARs), which act as metabolic sensors and regulators of lipid homeostasis [148].

AMPK pathway

**Figure 1:**
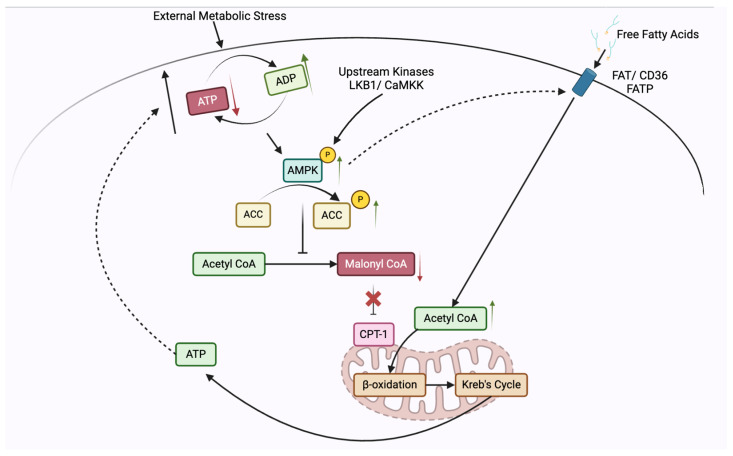
Fatty acid oxidation and Calorie Restriction. When CR activates AMPK, it enhances the phosphorylation of acetyl-CoA carboxylase (ACC), which inhibits malonyl-CoA synthesis. Malonyl-CoA is a key regulator of fatty acid synthesis, and its reduction relieves inhibition on carnitine palmitoyltransferase 1 (CPT1), allowing fatty acids to enter mitochondria for β-oxidation. This shift toward fatty acid oxidation reduces lipid accumulation, which is often associated with cancer progression, and limits the availability of substrates required for membrane biogenesis in tumor cells. Red arrows indicate down-regulation, while green arrows upregulation.

AMPK is a central energy sensor that becomes activated during conditions of low energy availability, such as CR. Upon activation, AMPK promotes catabolic pathways, including fatty acid oxidation, while inhibiting anabolic processes like lipogenesis [149]. In the context of cancer prevention, the activation of AMPK plays a pivotal role in promoting cellular resilience against oncogenic stressors. Metabolic challenges such as nutrient deprivation or energy stress, which are common during calorie restriction or fasting, increase the AMP/ATP ratio, leading to AMPK activation. Once activated, AMPK phosphorylates and inhibits acetyl-CoA carboxylase (ACC), reducing the synthesis of malonyl-CoA. This reduction relieves the inhibition on carnitine palmitoyltransferase-1 (CPT-1), allowing for enhanced mitochondrial import and oxidation of fatty acids (Figure 1).

By increasing fatty acid oxidation, AMPK shifts the cell’s metabolism from anabolic, energy-consuming processes (such as lipid and protein synthesis) to catabolic, energy-producing pathways. In the context of cancer, this metabolic shift deprives tumor cells of the lipid substrates needed for rapid membrane synthesis, which is essential for their unchecked proliferation [150]. Moreover, increased mitochondrial fatty acid oxidation generates ATP to meet the cell’s energy demands while reducing reliance on glycolysis, a pathway frequently hijacked by cancer cells to support their growth [151,152]. Additionally, by promoting fatty acid oxidation and reducing lipid accumulation, AMPK activation helps limit lipid-induced oxidative stress and inflammation—two key factors in cancer development. This metabolic reprogramming enhances the ability of cells to cope with oncogenic stressors, helping to suppress tumor initiation and progression [152]. Thus, through the regulation of lipid metabolism and energy homeostasis, AMPK activation contributes to cellular defense mechanisms that mitigate the risk of cancer.

Further, this shift reduces the availability of fatty acids for membrane synthesis, an essential process for rapidly proliferating cancer cells. Unlike normal cells, cancer cells rely heavily on de novo lipid synthesis to fuel their rapid growth and proliferation. By activating AMPK, CR disrupts this metabolic preference, redirecting cells away from anabolic processes such as lipogenesis and promoting catabolic pathways, particularly fatty acid oxidation [153]. Once activated under CR conditions, AMPK inhibits acetyl-CoA carboxylase (ACC), a key enzyme in fatty acid synthesis. This inhibition reduces malonyl-CoA levels, relieving its suppression on carnitine palmitoyltransferase-1 (CPT-1), which subsequently facilitates the transport of fatty acids into the mitochondria for oxidation. By favoring fatty acid oxidation over lipid storage, AMPK depletes cancer cells of the lipid precursors they require for membrane synthesis and energy production, creating a metabolic environment less favorable for tumor growth [154]. Moreover, in the context of CR, AMPK activation serves as a protective mechanism against the accumulation of lipid byproducts, such as lipid droplets and reactive oxygen species (ROS), both of which can exacerbate oncogenic signaling pathways [153]. By enhancing lipid catabolism, AMPK not only curtails the lipid reserves available for tumor cell expansion but also reduces oxidative stress and inflammation—two major contributors to cancer progression [155]. In this way, AMPK-mediated lipid catabolism under CR does not just support energy balance; it enforces metabolic constraints on cancer cells, limiting their access to key resources necessary for sustained proliferation. This shift helps to suppress tumor growth by depriving cancer cells of the metabolic flexibility they depend on, highlighting AMPK’s integral role in the anti-tumor effects of calorie restriction.

##### Sirtuin 1 (SIRT1) Pathway

CR influences key molecular regulators of metabolism and cellular stress response such as SIRT1, a NAD+-dependent deacetylase that plays a crucial role in extending the cellular lifespan, enhancing DNA repair, and promoting metabolic homeostasis. Under conditions of nutrient scarcity, such as during CR, the increase in the NAD+/NADH ratio activates SIRT1, which orchestrates a variety of adaptive responses aimed at maintaining cellular integrity and metabolic balance. This activation is particularly important in the context of cancer suppression, as SIRT1 modulates critical pathways involved in cell survival, proliferation, and stress resistance [156,157].

**Figure 2:**
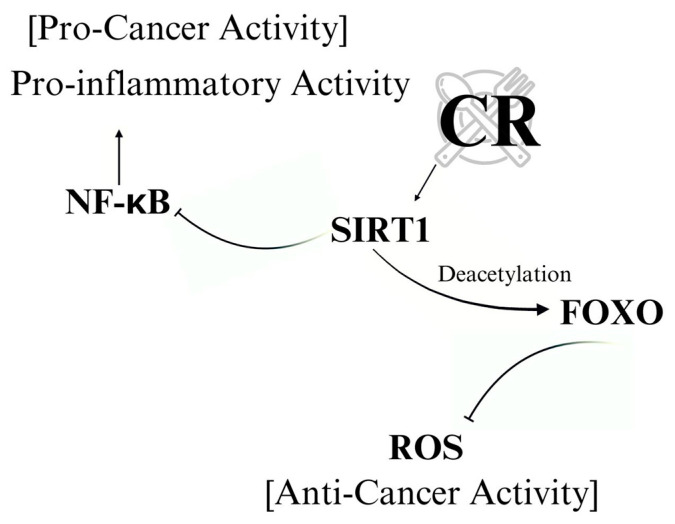
SIRT1 promotes anti-cancer activity under CR by inhibiting inflammatory pathways, such as the NF-κB signaling pathway. It also deacetylates and activates FOXO, thereby promoting cellular defenses against ROS and furthering anti-cancer activity.

Through its deacetylase activity, SIRT1 targets key transcription factors and proteins that regulate tumor suppressor pathways, including p53, FOXO, and PGC-1α. In cancer cells, SIRT1 activation enhances genomic stability by promoting DNA repair mechanisms, reduces oxidative stress by improving mitochondrial function, and induces apoptosis in damaged or pre-cancerous cells. By coordinating these cellular responses, SIRT1 functions as a guardian against tumorigenesis, aligning its anti-cancer effects with the metabolic adaptations induced by CR. Thus, the interplay between calorie restriction and SIRT1 not only supports cellular longevity and stress resistance but also acts as a metabolic barrier to cancer progression (Figure 2). For example, SIRT1 deacetylates and represses p53, a crucial tumor suppressor protein involved in DNA damage response, allowing for a balanced cellular outcome between DNA repair and apoptosis [158].

FOXO transcription factors, known for their role in oxidative stress resistance, cell cycle regulation, and apoptosis, are also key targets of SIRT1. By deacetylating FOXO, SIRT1 enhances cellular defenses against ROS, helping maintain genomic integrity and prevent cancer development (Figure 2). In addition, SIRT1 promotes mitochondrial function by activating PGC-1α, a master regulator of mitochondrial biogenesis. By improving mitochondrial efficiency and reducing ROS production, SIRT1 contributes to a healthier cellular environment, which is crucial for limiting cancer cell proliferation [159].

SIRT1 also suppresses inflammatory pathways, such as the NF-κB signaling pathway, which is often upregulated in cancer. Chronic inflammation is a major contributor to tumorigenesis, and by inhibiting NF-κB, SIRT1 reduces the pro-inflammatory conditions that can drive cancer development. The cumulative effect of SIRT1 activation under CR is a coordinated cellular response that enhances DNA repair, reduces inflammation, improves mitochondrial function, and eliminates damaged cells, thereby creating a less favorable environment for tumor growth [160] (Figure 2).

Thus, SIRT1 serves as a critical mediator of the anti-tumor effects of calorie restriction, integrating metabolic adaptations with cancer prevention by regulating lipid metabolism, inflammation, and cellular stress responses.

##### Mechanistic Target of Rapamycin (mTOR) Pathway

mTOR is a central regulator of cellular growth, proliferation, and metabolism. Under normal conditions, mTOR senses nutrient availability—specifically glucose, amino acids, and growth factors—and promotes anabolic processes such as protein synthesis and lipid production to support cell growth and division. However, dysregulation of the mTOR pathway is commonly observed in many cancers, where hyperactivation of mTOR drives uncontrolled cellular proliferation, enhances metabolic activity, and reduces autophagy, thereby contributing to tumorigenesis [161].

**Figure 3:**
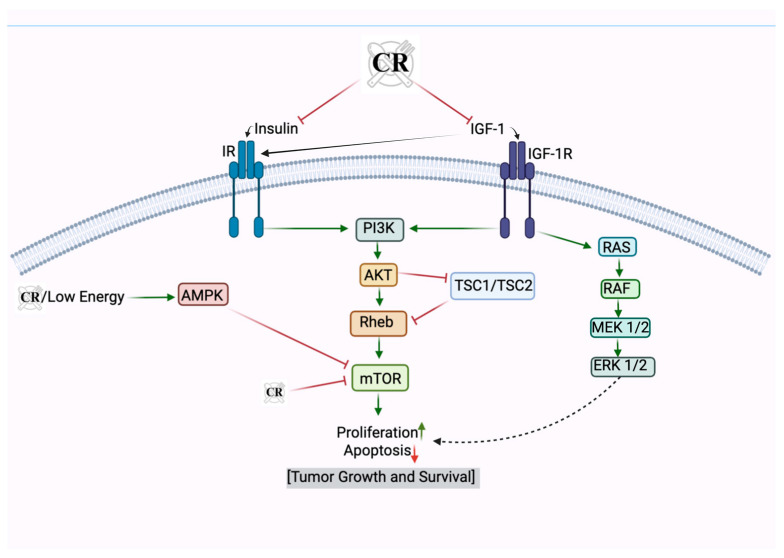
Overview of the impact of CR on different nutritional pathways indicating how it promotes anti-cancer activity. Red arrows represent downregulation, while green arrows indicate upregulation. Created in https://BioRender.com (accessed on 18 January 2025).

During periods of nutrient scarcity, such as under CR conditions, mTOR activity is inhibited. This inhibition occurs primarily through the activation of upstream regulators such as AMPK, which acts as a metabolic checkpoint under low-energy conditions (Figure 3). AMPK activation leads to the suppression of mTOR via the phosphorylation of TSC2, a component of the tuberous sclerosis complex that negatively regulates mTOR activity [161,162].

By downregulating mTOR signaling, CR induces a metabolic shift that favors catabolic pathways, such as autophagy and fatty acid oxidation, while reducing anabolic processes like protein synthesis. This shift helps reduce the accumulation of damaged proteins and organelles, enhance cellular quality control mechanisms, and promote genome stability. Autophagy, in particular, plays a crucial role in cancer prevention by clearing damaged organelles and proteins that, if accumulated, could trigger oncogenic signaling [161]. Inhibition of mTOR by CR also promotes cellular quiescence, limiting excessive proliferation—a hallmark of cancer. By slowing down cell cycle progression, mTOR inhibition reduces the metabolic demands on cells, making it more difficult for pre-cancerous or cancerous cells to meet the biosynthetic requirements necessary for rapid division and growth. Furthermore, the suppression of mTOR under CR conditions enhances apoptosis in cancer cells, promoting the selective elimination of cells with DNA damage or oncogenic mutations [83]. Additionally, mTOR inhibition reduces the activity of pathways linked to angiogenesis and inflammation, both of which are critical for tumor growth and progression. By suppressing the synthesis of pro-inflammatory cytokines and growth factors, CR-mediated mTOR inhibition creates a less favorable microenvironment for tumor initiation and invasion [83].

##### Insulin-like Growth Factor 1 (IGF-1) Pathway

IGF-1, a hormone primarily produced in the liver in response to growth hormone stimulation, activates the IGF-1 receptor (IGF-1R) on the surface of cells. This activation triggers downstream signaling cascades such as the PI3K/AKT and RAS/RAF/MEK/ERK pathways, both of which promote cell proliferation, inhibit apoptosis, and stimulate anabolic processes like protein synthesis—all of which are critical for tumor growth and survival [163]. In cancer, the IGF-1 pathway plays a crucial role in creating an environment conducive to tumor development by driving cell division and inhibiting apoptosis. High levels of IGF-1 have been associated with increased risks of various cancers, including breast, prostate, and colorectal cancers.

CR significantly lowers the circulating levels of IGF-1, thereby attenuating the activation of IGF-1R and its downstream pro-tumorigenic pathways. This results in decreased cellular proliferation and increased apoptosis in pre-cancerous and cancerous cells [164]. Both studies in animals and humans demonstrated that reduction in the circulating IGF-1 levels by CR suppresses the activity of the PI3K/AKT pathway. This suppression diminishes the availability of survival signals for cancer cells, promoting apoptosis and reducing the potential for tumor growth (Figure 3) [164]. Moreover, by decreasing IGF-1 signaling, CR enhances cellular stress resistance and genome stability. Lower IGF-1 levels shift cells into a more quiescent state, reducing unnecessary proliferation, thereby lowering the risk of accumulating mutations that could lead to cancer. Additionally, by reducing anabolic signals, CR decreases the metabolic demands on cells, reducing oxidative stress and limiting DNA damage—a key driver of tumorigenesis [164].

Furthermore, lower IGF-1 levels result in reduced angiogenesis, the process through which new blood vessels form to supply nutrients and oxygen to tumors. Without the support of increased angiogenesis, tumors are less able to grow and invade surrounding tissues. CR-mediated IGF-1 reduction also suppresses inflammation, another key contributor to cancer progression, by reducing the activation of pro-inflammatory pathways like NF-κB, which are often stimulated by IGF-1 signaling [164].

Evidence indicates that the growth hormone (GH)-IGF-1 axis plays a significant role in cancer incidence and progression, supported by epidemiological studies linking elevated plasma IGF-1 levels to increased cancer risk and the observed absence of cancers in individuals with GH/IGF-1 deficiency [165].

Insulin promotes cellular glucose uptake and stimulates signaling pathways such as PI3K/AKT/mTOR, which enhances cell growth, proliferation, and survival [166]. IGF-1, a key regulator of growth and development binds to the IGF-1 receptor (IGF-1R) and activates downstream signaling cascades, including the PI3K/AKT/mTOR [167] and RAS/RAF/MEK pathways [168]. Chronic hyperinsulinemia, often linked with obesity and insulin resistance, creates a pro-growth environment that can promote tumorigenesis.

Since CR leads to improved insulin sensitivity and lower circulating insulin levels, as well as decreased IGF-1 levels and reduced activation of its receptor, it has been known to decrease the activation of pro-growth pathways associated with insulin signaling and mitigate the mitogenic and anti-apoptotic effects of IGF-1.

The effect of CR on various nutritional pathways and how it promotes anti-cancer activity is summarized in Figure 3.

#### 3.1.2. CR: Clinical and Epidemiological Evidence

Considering the promising effects associated with CR-based interventions in vitro and in preclinical models, scientists also wanted to ascertain CR’s effects on cancer prevention in clinical studies. To date, only a limited number of clinical studies have been able to show CR’s potential as a preventive approach against cancer development.

However, it is important to point out that CR without malnutrition, in humans, ameliorates multiple metabolic and hormonal factors, similar to those observed in rodent models, that are implicated in the pathogenesis of cancer [169,170]. In fact, reduced concentrations of circulating inflammatory markers (such as C-reactive protein—CRP, TNF-α and IL-6); ameliorated insulin sensitivity; increased serum concentrations of Insulin-like Growth Factor Binding Protein 1 (IGFBP-1) and 2 (IGFBP-2); augmented circulating levels of adiponectin; increased anti-cancer immunity; reduced leptin and the leptin-to-adiponectin ratio; and decreased bioavailable testosterone and estrogen have been observed [164,171,172,173]. Moreover, CR acts on several processes known to be involved in the pathogenesis of cancer, being able to reduce oxidative stress, free radical-induced DNA damage, cell proliferation and cell senescence biomarkers; enhance DNA repair processes; increase damaged cell removal via apoptosis; and boost autophagy and mechanisms of protection against toxins [164,171].

Most human studies providing evidence of CR’s role in cancer prevention are retrospective. These studies often involve populations that experienced calorie restrictions due to factors such as war, famine, or specific cultural dietary practices. For instance, some historical circumstances seemed to indicate that CR could be effective in human cancer prevention. Prepuberal girls who were exposed to the Norwegian famine in World War II (consuming an average of 22% fewer calories than the expected amount) had a lower rate of breast cancer than women from earlier or later birth cohorts [174]. Research conducted in Denmark showed that patients affected by anorexia nervosa (AN), from 1970 to 1993, had a lower cancer risk, compared to the general population [175]. Moreover, a retrospective study of around 15,000 women with AN found a lower risk of breast cancer but an increased risk of other tumors [176]. This highlights that dietary restriction without malnutrition is key to achieving beneficial effects. Further direct evidence comes from an 11-year study primarily focused on weight management, where participants underwent an intensive lifestyle intervention. This included restricting calorie intake to 1200–1800 kcal per day and engaging in moderate-intensity physical activity weekly, which resulted in a lower incidence of obesity-related cancers [177]. Further, a two-year study on CR in humans demonstrated a reduced metabolic rate and a decrease in systemic oxidative stress markers, underscoring CR’s potential for health benefits [178].

More recently, a Swedish study also demonstrated that women hospitalized for AN before the age of 40 years had a 53% lower incidence of BC than the general female population [179]. Both CR and anorexia can reduce estrogen and IGF-1 levels [179,180]; the latter has been identified as a biomarker for BC prediction [181,182]. Finally, AN is very often associated with amenorrhea, thus resulting in a decreased number of lifetime ovulations, which might also lower the risk of BC incidence [183]. A very recent retrospective cohort study also showed low rates of BC and of all cancers combined in English women hospitalized with AN (from 1999 to 2021) [176]. Some epidemiological retrospective evidence seems to also indicate CR involvement in human cancer occurrence prevention: a reduced incidence of BC has been found in women of the Okinawa community, whose traditional diet was low in calories, compared to the other Japanese women [184]. Moreover, the Okinawa community showed a decreased prevalence of cancer, which has been considered, together with the low incidence of vascular diseases, to be accountable for their tumor-free longer life and overall lower mortality rate [185].

Besides those historical and epidemiological observations, some recent controlled studies also indicated a promising effect of CR on cancer prevention. A recent Spanish multicenter matched case–control study, analyzing the association of excessive energy intake and CR with BC risk, demonstrated that premenopausal women consuming few calories (>20% below the expected amount) had a lower BC risk, while postmenopausal women with an excessive caloric intake (≥40% above the expected amount) showed an increased risk. Moreover, for every 20% increase in relative caloric intake, the probability of developing a hormone-receptor-positive or a HER2+ tumor increased by 13%, while the risk of developing a triple-negative tumor rosed by 7% [186]. When comparing the dietary intakes of BC patients with healthy controls, scientists showed that a high caloric intake raised cancer risk for all hormone-receptor-positive tumors, CR (>20% below the expected value of calories) instead exerted a protective effect [186].

Other observational studies have shown that weight loss via CR reduces the risk of postmenopausal BC [172,187]. Moreover, a randomized trial that enrolled premenopausal participants demonstrated that both intermittent and continuous CR, during 6 months, not only resulted in weight loss of overweight and obese women, but also improved several cancer risk biomarkers (reduction in leptin, free androgen index, high sensitivity CRP, total and low-density lipoprotein (LDL) cholesterol, triglycerides, blood pressure, and increase in sex hormone binding globulin, IGFBP-1, and 2) [188]. Several other studies, performed on both premenopausal and postmenopausal women, have also demonstrated a reduction in biomarkers of estrogen stimulation and inflammation [189].

Despite some interesting data, it has to be taken into account that CR has issues of compliance and possible side effects [190]; hence, it could hardly be applied and consistently recommended to the general population. Nevertheless, as already stated, other standardized and well-tolerated dietary regimens, with few or no adverse effects, namely, FMD and IF, have been identified and started to be evaluated as cancer-preventive approaches, even in clinical studies.

### 3.2. Proposed Mechanisms and Evidence for the Anti-Tumor Activity of Fasting-Mimicking Diet (FMD)

FMD is a dietary approach designed to imitate the metabolic effects of PF while still allowing some food intake (Table 1). FMD offers the metabolic benefits of fasting without requiring extended periods of complete calorie restriction, making it a more feasible option for cancer prevention and treatment [112].

#### 3.2.1. FMD: Cellular and Molecular Effects on Nutrient-Sensing Pathways

FMD may help prevent cancer, possibly by acting on metabolic pathways such as reducing IGF-1, activating AMPK, and/or affecting nutrient-sensing mechanisms that play a critical role in regulating cell proliferation, death, and survival.

##### Insulin-like Growth Factor 1 (IGF-1) Pathway

A critical mechanism through which FMD exerts its anti-cancer effects is the reduction in circulating IGF-1. IGF-1 plays a vital role in promoting cell proliferation and inhibiting apoptosis, both of which are processes that can drive tumorigenesis. Elevated IGF-1 levels have been linked to increased cancer risk, particularly for cancers such as breast, prostate, and colon cancers (see Section 2.3.1).

FMD reduces IGF-1 signaling by mimicking the nutrient deprivation seen in fasting. This reduction in IGF-1 levels leads to decreased activation of the PI3K/AKT and RAS/RAF/ERK pathways, which are crucial for cancer cell survival and proliferation. Preclinical studies have shown that periodic cycles of the FMD significantly lower IGF-1 levels, making tumor cells more susceptible to stress and less capable of resisting apoptosis [191].

##### Mechanistic Target of Rapamycin (mTOR) Pathway

Another key pathway influenced by the FMD is the mTOR pathway, a master regulator of cell growth and metabolism. In cancer, mTOR is often hyperactivated, leading to uncontrolled cell growth and survival. FMD inhibits the mTOR pathway by inducing a state of perceived nutrient deprivation, much like traditional fasting.

The inhibition of mTOR results in a metabolic shift away from anabolic processes such as protein synthesis and cell proliferation toward catabolic processes, including autophagy. This process allows cells to degrade and recycle damaged components, which helps prevent the accumulation of cellular damage that can drive tumorigenesis. The suppression of mTOR by the FMD not only limits the growth of cancer cells but also enhances their sensitivity to chemotherapy by reducing their ability to repair damage [192].

##### AMP-Activated Protein Kinase (AMPK) Pathway

The AMPK pathway is a central energy sensor activated during periods of low energy availability, such as during fasting or following an FMD cycle. Activation of AMPK leads to the inhibition of mTOR and the promotion of fatty acid oxidation and glucose uptake, creating an unfavorable environment for cancer cell growth.

AMPK activation by the FMD promotes autophagy, a process that is critical for maintaining cellular homeostasis under stress conditions. In cancer cells, which rely heavily on anabolic pathways to support their rapid growth, this shift toward autophagy and metabolic stress can lead to apoptosis. Additionally, AMPK activation can suppress the inflammatory NF-κB signaling pathway, which is often upregulated in cancer, reducing inflammation-driven tumor progression [192].

#### 3.2.2. FMD: Clinical and Epidemiological Evidence

As prolonged CR may be challenging for humans, alternative regimens more suitable to be followed, such as FMD, have been tested in clinical trials, to analyze their effects on health, even though quite a substantial amount of preliminary human data already indicate this regimen could only modulate many cellular mechanisms and pathways involved in cancer development.

A pilot clinical trial, performed on healthy adults, showed it to be feasible, without any major adverse effects, and able to decrease some biomarkers for cancer, namely, reducing fasting blood glucose levels and circulating IGF-1 [110]. A subsequent randomized controlled trial demonstrated that FMD reduced body weight and body fat, decreased blood pressure, upregulated IGFBP-1 and ketone bodies, and lowered IGF-1 levels, glucose, triglycerides, cholesterol, and CRP [112]. Together, these data suggest that FMD in healthy humans might prevent obesity and inflammation-related pathologies, such as cancer.

### 3.3. Proposed Mechanisms and Evidence for the Anti-Tumor Activity of Intermittent Fasting (IF)

Fasting, which includes IF and PF, has gained significant attention as a potential strategy for cancer prevention and treatment in recent years (Table 1). The body undergoes a metabolic shift from reliance on glucose to fatty acid oxidation and ketone body production during fasting, creating an environment that is less favorable for cancer cell proliferation [193]. The effects of fasting on cancer are mediated by several key molecular pathways, including the IGF-1, mTOR, AMPK, and autophagy pathways, each of which plays a distinct role in regulating metabolism, cell growth, and survival under nutrient-deprived conditions. Both PF and IF have an impact on metabolic pathways (such as IGF-1, mTOR, AMPK, and autophagy), but the specific effects can vary depending on the duration and frequency of fasting [194].

- IF typically involves shorter periods of fasting (e.g., 16–48 h) on a regular basis (e.g., ADF, TRE, or TRF) (Table 1). IF has been shown to positively influence the same pathways as PF by reducing IGF-1 levels, inhibiting mTOR, activating AMPK, and promoting autophagy during the fasting periods. In IF, these changes are cyclic and occur during the fasting phase but are partially reversed during the feeding phase, leading to repeated metabolic stress for cancer cells [193].
- PF usually refers to extended fasting periods lasting several days (e.g., 48–72 h), with significant effects on the same pathways (Table 1). During PF, the metabolic shift to ketone body production becomes more pronounced, and the inhibition of IGF-1 and mTOR, as well as the activation of AMPK and autophagy, are sustained for longer periods. This more extended nutrient deprivation can further stress cancer cells and may enhance tumor suppression mechanisms more effectively.

While both types of fasting target cancer-related pathways, the intensity and duration of pathway activation or inhibition can differ. IF induces cyclical effects, while PF leads to more sustained alterations in metabolic and cellular homeostasis, leading to lasting biological changes [194].

#### 3.3.1. IF: Cellular and Molecular Effects on Nutrient-Sensing Pathways

IF has emerged as a promising approach for modulating key cellular pathways involved in metabolism, aging, and disease prevention. By imposing cycles of fasting and feeding, IF influences nutrient-sensing pathways, which play essential roles in maintaining cellular health and resilience against stress. This metabolic reprogramming involves several critical pathways, including AMPK, which boosts energy efficiency; the mTOR pathway, known for regulating growth and autophagy; and the IGF-1 pathway, impacting cellular proliferation. Additionally, IF stimulates autophagy, enhancing stress resistance, and promotes ketogenesis, reducing oxidative stress.

Together, these mechanisms underscore IF’s potential in promoting metabolic health and protecting against cancer and other age-related diseases [195,196,197].

##### AMP-Activated Protein Kinase (AMPK) Pathway

AMPK is a critical energy sensor that is activated during fasting in response to increased AMP/ATP ratios. When energy levels are low, as in fasting, AMPK activation inhibits mTOR signaling and promotes the uptake of glucose and fatty acids for energy production via fatty acid oxidation. This metabolic shift supports normal cell function under nutrient deprivation but creates stress in cancer cells, which are heavily dependent on glucose and other anabolic processes for their rapid growth [198].

Activation of AMPK by fasting also enhances autophagy and inhibits protein synthesis via mTOR suppression, further reducing the growth and survival of cancer cells. Additionally, AMPK activation promotes apoptosis in cancer cells by stabilizing p53, a tumor suppressor protein, and inhibiting cancer-promoting pathways such as NF-κB. Therefore, fasting, through AMPK activation, reinforces metabolic stress on cancer cells while preserving normal cell function [199,200].

##### Mechanistic Target of Rapamycin (mTOR) Pathway

The mTOR pathway is a central regulator of cell growth and metabolism, responding to nutrient availability, particularly amino acids and glucose. Fasting inhibits mTOR activity due to nutrient depletion, leading to a shift away from anabolic processes like protein and lipid synthesis toward catabolic processes such as autophagy. Cancer cells, which often rely on mTOR hyperactivation to support their rapid proliferation, are particularly vulnerable to mTOR inhibition induced by fasting.

Inhibition of mTOR by fasting also reduces cellular proliferation and promotes autophagy, a process through which damaged cellular components are degraded and recycled. This autophagic process is crucial for maintaining cellular homeostasis and protecting against tumorigenesis by clearing damaged organelles, misfolded proteins, and potentially oncogenic mutations. The suppression of mTOR during fasting is therefore a key mechanism through which fasting exerts anti-cancer effects [201].

##### Insulin-like Growth Factor 1 (IGF-1) Pathway

One of the most well established effects of fasting is a significant reduction in circulating levels of IGF-1, which plays a crucial role in promoting cellular proliferation and inhibiting apoptosis, with elevated levels of IGF-1 being linked to increased cancer risk [202]. Fasting reduces IGF-1 signaling, leading to decreased activation of the PI3K/AKT and RAS/RAF/ERK pathways, which are critical for tumor cell survival and growth. By downregulating IGF-1, fasting creates a metabolic environment where cancer cells are more susceptible to apoptosis. Moreover, lower IGF-1 levels lead to reduced angiogenesis and inflammation, two processes that support tumor progression. Unsurprisingly, preclinical studies on breast, colon, and prostate cancer models have demonstrated that fasting protocols, by reducing IGF-1 signaling, effectively limit tumor growth as well [15].

##### Autophagy and Stress Resistance

Autophagy is a catabolic process that is upregulated during fasting as a survival mechanism in response to nutrient scarcity. During fasting, the inhibition of mTOR and activation of AMPK promote autophagy, allowing cells to recycle damaged organelles and proteins to maintain energy homeostasis [201]. This process is especially important for cancer prevention as it inhibits the accumulation of cellular damage and mutations that could lead to cancer.

In cancer cells, which often rely on autophagy to survive under stress conditions, fasting-induced autophagy can have a dual effect. While autophagy initially helps cancer cells survive nutrient deprivation, prolonged activation of autophagy can lead to cell death by depleting cellular resources. Fasting thus induces a metabolic state that selectively impacts cancer cells, pushing them toward autophagic or apoptotic death, while normal cells remain protected [200,201].

##### Ketogenesis and Oxidative Stress

During fasting, the body shifts from glucose metabolism to fatty acid oxidation, leading to the production of ketone bodies. Cancer cells, which predominantly rely on glucose for their energy needs through aerobic glycolysis (the Warburg effect), are less able to utilize ketone bodies for energy. This metabolic shift places additional stress on cancer cells, which struggle to adapt to the low-glucose, high-ketone environment induced by fasting [196,197,203].

Moreover, fasting reduces the levels of ROS, which are often elevated in cancer cells due to their increased metabolic activity. By reducing oxidative stress, fasting limits the DNA damage and mutations that can drive tumorigenesis. At the same time, fasting enhances the resilience of normal cells to oxidative stress by promoting DNA repair and activating stress response pathways, further protecting them from cancerous transformation [204].

#### 3.3.2. IF: Clinical and Epidemiological Evidence

Similarly to FMD, IF has been proposed as an alternative to chronic CR, and, to date, a few clinical studies have shown its potential as a cancer preventive measure. In fact, although there are no direct studies linking IF to reduced cancer risk in humans, its effect has been explored through its impact on tumor-related factors, such as insulin, glucose [205], IGF-1 [206], leptin levels [207], adiponectin [208] and weight loss [202,209,210,211]. Fasting glucose levels, which can be improved by IF [212], appear to be a significant risk factor for cancer, as demonstrated by a population cohort study. Individuals with persistently high, uncontrolled glucose levels over several years may, in fact, face an increased risk of developing cancer [205]. However, the findings on IF’s effect on these cancer risk markers have been mixed. Initial small-scale studies showed that TRF positively influenced weight loss and related physiological parameters, such as insulin sensitivity [213,214,215]. However, larger studies failed to replicate these effects, showing no significant impact on fasting insulin or on inflammatory C-reactive protein [80,87,88] with either TRF or ADF.

Other trials indicated that 5:2 and ADF regimens might modulate some cancer risk factors implicated in tumor pathogenesis. Hence, they are able to reduce fasting glucose, insulin, and leptin, and augment adiponectin and ketone levels [213,216,217]. In agreement with that, 50 healthy volunteers (29 women and 21 men) who followed IF showed a reduction in body weight, body fat, systolic and diastolic blood pressure, as well as a decline in proinflammatory cytokines IL-1β, IL-6 and TNF-α [218].

Other studies demonstrated that IF interventions are able to confer humans several health benefits, probably not simply depending on the caloric intake reduction itself, such as improvement of dyslipidemia, insulin resistance, obesity, hypertension, and inflammation [178]. A clinical trial conducted on 16 healthy participants who followed an ADF regimen for 22 days showed that they lost 2.5% of their original weight and 4% of fat mass, together with a 57% drop in fasting insulin levels [219]. Interestingly, when comparing two groups of overweight women assigned to either a 5:2 regimen or a 25% reduction in daily caloric intake for 6 months, even though the same amount of weight was lost in both groups, those following the 5:2 diet experienced a greater decrease in waist circumference and obtained a bigger improvement in insulin sensitivity [188,220].

Some recent works also described a noteworthy reduction in oxidative stress markers after TRF [221,222,223] and ADF [217].

Interestingly, a study conducted on people at high risk of breast cancer demonstrated that, after a month of IF, all subjects experienced 4.8% weight and 8% fat loss, as well as an amelioration of insulin resistance [224]. Moreover, one case–control study suggested that prolonged overnight fasting and early breakfast may be associated with a lower risk of prostate cancer [225].

Even though the effects of IF on tumor incidence remain unanswered, preliminary emerging evidence suggests that those dietary regimens may have the potential to lower cancer risk by regulating body weight, boosting normal cell stress resistance, and modulating specific metabolic and molecular pathways. Therefore, more clinical studies are needed to clarify the outcomes of IF on human health so that those regimens could possibly be standardized and employed as other dietetic cancer-preventive measures.

### 3.4. Effects of CR and Fasting on Microbiota

Up to 100 trillion symbiotic microorganisms, including bacteria, fungi, parasites, and viruses, colonize the gastrointestinal (GI) tract, encompassing 10 times the number of cells in the body itself. This internal community, which is mainly composed of bacterial cells (~99%), is called the gut microbiota [226]. The microbiota engages a relentless crosstalk with the intestinal epithelium, serving a pivotal role in many host physiological activities (energy harvest, metabolism, immune response, immune system maturation, regulation of neurological and cognitive development, etc.) and pathological processes [227]. Indeed, gut microbiota dysbiosis can lead to conditions such as overweight, obesity, type 2 diabetes, inflammatory bowel disease (IBD), irritable bowel syndrome (IBS) and even colorectal cancer (CRC) [228,229]. How exactly gut microbiota influences CRC susceptibility and progression is still mainly unknown [230,231]; however, it is possible that it works by affecting immune system activity, and through mechanisms such as inflammation and DNA damage, as well as by excreting chemicals able to either promote or inhibit tumor formation [232].

Mounting evidence shows that diet is among the most critical factors in modulating microbiota composition, regulating, in turn, the host physiological and pathological responses. In fact, dietary components can alter immunological and inflammatory parameters of gut microbiota composition, and regulate tissue inflammation, cancer initiation, and/or progression. For instance, it has been proven that the typical “Western Diet (WD)”, high in fat, sugars, and animal products, not only contributes to the global increase in obesity but also causes microbiota dysbiosis, alterations in intestinal immune cell homeostasis, and perturbation of the barrier integrity. In contrast, traditional dietary patterns, such as the Mediterranean diet, negatively correlate with serum markers of inflammation, overall exerting beneficial effects on gut microbiota [57,233,234,235,236,237,238,239].

Indeed, exploring how DR might modify and influence the microbes in the gut emerges as a very stimulating subject. Albeit interesting and growing, the evidence on this topic is still slightly limited and mainly restricted to animal studies.

In recent years, many studies analyzed the impact of DR programs on gut microbiota composition, both in animal and human models, identifying changes in microbial diversity, namely, modifications in the abundance and type of microbial inhabitants [240,241,242,243,244,245,246,247,248,249,250,251]. Those changes have been connected with specific metabolic modifications [240,246,252,253,254]. Differences in gut microbiota composition, in fact, correlate with host wellbeing; thus, CR and fasting might contribute, for instance, to maintaining and re-establishing gut barrier permeability (potentially enhancing anti-inflammatory responses in many bowel inflammatory diseases); regulating lipid metabolism, glucose tolerance, insulin sensitivity, body weight and fat mass equilibrium; exerting an immune-modulation activity; decreasing the inflammatory status; and even modulating brain development, cognitive functions and age-related cognitive decline [240,255,256,257,258,259]. Several significant studies on those topics, published until 2021, have been reviewed here [260,261]; therefore, in the next section, we will briefly describe a few of the most noteworthy and recent studies connecting DR interventions with microbiota composition, host homeostasis, and health.

Preclinical studies conducted in animals, either healthy or affected by some kind of pathology (obesity, colitis, etc.), indicate that different fasting regimens are able to modulate microbiota dynamics and their impact on the health status. For instance, a TRF program (once-a-day feeding for 28 weeks) significantly lowered the alpha diversity of rats’ microbiota, decreased Actinobacteria and Patescibacteria richness, and amplified Verrucomicrobia abundance [262]. Healthy rats who followed a 5-week IF (18:6) intervention augmented alpha diversity, while the Firmicutes/Bacteroidetes ratio was significantly decreased compared to the control group [263]. Transplantation of gut microbiota from an obese woman, before and after an 8-week very-low-calorie diet (800 kcal/day), into germ-free mice increased their alpha diversity, decreased the abundance of several microbial taxa, known to be overrepresented in obese humans, reduced animal body fat accumulation, and improved their glucose tolerance compared to controls; finally, this dietary restriction induced a shift towards the naïve T and B cell compartment, thus delaying immune senescence [264]. In a xenograft BALB/c male nude mouse model, CR (animals were fed with 70% of the usual food intake) effectively reduced CRC tumor volume and weight. In addition, CR modified microbiota composition, increasing the Lactobacillus constituent ratio, hence ameliorating microbial dysbiosis [265].

Overall, the most recent preclinical studies confirmed that gut microbiota composition is affected by DR interventions and indicated that many kinds of fasting can ameliorate metabolic disorders (including cancer, obesity, type 2 diabetes, and hypertension, as well as inflammatory and neurodegenerative diseases). However, the results are not always homogeneous when considering bacteria strain/group changes and dynamics; also, different DRs display diverse effects on microbiota, and changes in microbial diversity do not last forever. In conclusion, despite promising and interesting, data from preclinical studies are still extremely heterogenous and do not allow any general conclusions regarding the exact effects of all the variety of feeding restrictions on gut microbiota to be drawn yet.

The impact of different DRs on human microbiota was also investigated on healthy and unhealthy subjects. Different Ramadan-based studies performed on healthy individuals showed, overall, a substantial remodeling of the gut microbiota [251,266,267], sometimes associated with a positive impact on glucose level, lipid metabolism, insulin signaling, and anti-cancer serum proteomic signatures [268,269].

A TRF (16:8) intervention followed over 12 weeks by healthy men showed a significant change in microbial diversity: the Bacteroidetes phylum (potentially beneficial for the gut flora) increased in the TRF group compared to the control one [270]. Moreover, this change in microbiota richness was linked to SIRT1 activation [271].

A randomized clinical trial, conducted on patients with metabolic syndrome showed that 8 weeks of IF (5:2) significantly increased the relative abundances of short-chain fatty acid (SCFA) phyla producers and decreased the circulating levels of LPS compared to the control group [272].

A very-low-calorie diet (800 kcal/day) followed for 46 days by obese postmenopausal women showed an overall parallel shift in bacteria community structure, which correlates with fecal bile acid composition variations [273].

Eight weeks of calorie restriction (800–1200 kcal/day) in obese prediabetics patients determined significant changes in gut microbiota associated with weight loss [274].

Other studies tried to establish whether a common pattern of microbiota changes could be determined by different dietary restrictions. For instance, a randomized controlled study compared the effects of intermittent CR (the participants reduced calorie intake by ~75% on 2 non-consecutive days of the week—5:2 diet) versus constant CR (the participants reduced their daily calorie intake by 20%) on the fecal microbiota of 147 obese adults. With the exception of Lactobacillales, which was enriched after intermittent CR, microbiome composition did not notably diverge between the groups. However, both diets resulted in a ~5% weight loss, associated with significant metabolic improvements, and correlations between insulin sensitivity and Akkermansiaceae, Christensenellaceae, and Tanerellaceae were found, highlighting the possible role of those bacteria in modulating host metabolic status [275].

A real-life study, comparing the effects of a 12-week TRF (<12 h feeding) with a CR (500–1000 kcal/day) in patients with obesity, demonstrated that besides weight loss (median weight loss of 4.0 kg and 2.2 kg in TRF and CR groups, respectively), no differences in alpha and beta diversity, gut microbiota composition, or other anthropometric variables were detected. However, Lachnospiraceae, Parasutterella, and Romboutsia significantly augmented in the TRF group [276].

Interestingly, different kinds of DR clearly impact both animal and human microbiota taxonomic composition and metabolite production. However, due to the heterogeneity and limited number of the studies published to date, as well as microorganisms and individual response variability, the data are still insufficient to define a distinctive pattern of gut microbiota changes induced by each different dietary approach. This lack of understanding hinders the opportunity for scientists to first identify and then apply specific interventions to prevent or control the progression of certain pathologies, including cancer. Hence, further research is needed, especially from randomized controlled studies, to fill those knowledge gaps so that it could be possible, in the near future, to establish guidelines to use DRs for chronic disease prevention and even therapy.

### 3.5. Effects of CR and Fasting on Stem Cell Biology

Stem cells are integral to tissue homeostasis and regeneration, playing a critical role in both normal physiological processes and disease pathogenesis, including cancer. Emerging research highlights how CR and various forms of fasting, including IF and FMD, influence stem cell function, particularly by altering metabolic pathways, reducing oxidative stress, and promoting tissue regeneration. These effects may have significant implications for aging, tissue repair, and cancer prevention (Table 2) [277].

#### 3.5.1. Metabolic Reprogramming and Stem Cell Function

One of the key mechanisms through which CR and fasting impact stem cells is by inducing metabolic reprogramming. Under nutrient-rich conditions, stem cells tend to rely on glycolysis for energy production, a process that supports rapid proliferation. However, during periods of CR or fasting, a metabolic shift occurs, favoring oxidative phosphorylation (OXPHOS) and fatty acid oxidation over glycolysis. This metabolic switch enhances stem cell quiescence, a state in which stem cells are preserved in a dormant state, protecting them from environmental stress and cellular damage (Table 2) [278].

This metabolic adaptation is crucial for long-term stem cell maintenance. Studies show that fasting promotes the self-renewal of hematopoietic stem cells (HSCs) in the bone marrow, enhancing their regenerative capacity. In models of aging, fasting has been found to rejuvenate aged stem cells, restoring their ability to produce new, functional cells, and CR has been shown to preserve muscle stem cells (satellite cells), enhancing muscle regeneration following injury (Table 2). These suggest that by modulating metabolic pathways, CR and fasting protect stem cells from the damaging effects of aging and environmental stressors [279,280].

#### 3.5.2. CR Reduces Oxidative Stress-Induced DNA Damage in Stem Cells

Oxidative stress refers to an imbalance between the production of ROS and the body’s ability to detoxify these harmful molecules, leading to cellular damage and contributing to aging and disease. CR and fasting also exert protective effects on stem cells by reducing oxidative stress.

ROS are a byproduct of cellular metabolism and, when in excess, they can damage DNA, proteins, and lipids, accelerating aging and increasing the risk of cancer. Stem cells are particularly vulnerable to oxidative stress, given their long lifespan and capacity for self-renewal. Both CR and fasting lower ROS levels by reducing mitochondrial activity and shifting the metabolic balance toward pathways that generate less oxidative damage [281].

By lowering oxidative stress, CR and fasting not only protect stem cells from DNA damage but also promote their longevity and functional capacity (Table 2). This protective effect is particularly evident in the context of aging, where the accumulation of DNA damage in stem cells leads to a decline in tissue regeneration. For example, fasting has been shown to reduce oxidative damage in intestinal stem cells, improving intestinal function and reducing the risk of age-related diseases [282].

#### 3.5.3. CR Enhances Stem Cell Regeneration via Autophagy Activation

Both CR and fasting activated autophagy, a process that allows cells to degrade and recycle damaged organelles and proteins [282]. Autophagy is essential for maintaining cellular homeostasis, and in stem cells, it plays a critical role in preserving their regenerative potential. During periods of nutrient deprivation, such as fasting, autophagy is upregulated to eliminate damaged components, preventing the accumulation of cellular debris that can impair stem cell function [283,288]. This enhanced autophagic activity has been linked to improved stem cell regeneration in several tissues. For example, fasting stimulates the regeneration of neural stem cells (NSCs) in the brain, enhancing cognitive function and neurogenesis. Similarly, autophagy induced by CR promotes the survival and regeneration of muscle stem cells, contributing to improved muscle repair [278,279]. By promoting autophagy, CR promotes renewal and protects against the degenerative effects of aging and disease (Table 2).

#### 3.5.4. The Role of CR and Fasting on Cancer Stem Cells (CSCs)

The effects of CR and fasting on stem cell biology have significant implications for cancer prevention. CSCs, which share many characteristics with normal stem cells, are thought to be responsible for tumor initiation, progression, and resistance to therapy. By influencing key pathways that regulate both normal and CSCs, CR and fasting may reduce the risk of cancer development and enhance the efficacy of cancer treatments [153,284,289,290].

Fasting has been shown to selectively stress CSCs, making them more susceptible to chemotherapy while protecting normal stem cells from the toxic effects of this treatment. This dual effect is mediated by changes in nutrient-sensing pathways, such as mTOR and AMPK, which are differentially regulated in cancer and normal cells (Table 2). As a result, fasting may enhance the therapeutic window of cancer treatments, reducing side effects and improving disease outcomes [153,284]. The specific effects of CR or fasting on CSCs have been demonstrated in studies utilizing either CR or CR mimetics. For instance, CR mimetics have been shown to decrease breast-cancer-stem-cell-marker-expressing cells in a SIRT1-dependent manner in the MDA-MB231 breast cancer model [291]. Similarly, in the MMTV-ERBB2 model, both CR and CR mimetics were found to inhibit cancer stem cell populations, particularly luminal and mammary stem cell-enriched populations, as well as their self-renewal (Table 2) [98,285].

In contrast, CR appears to have a different effect in leukemia, where it increases a subset of cells resembling the CSC population, similar to its effects on normal stem cells [286]. A comparable phenomenon was observed in intestinal stem cells, where refeeding after fasting was associated with an increase in their number and tumorigenic potential (Table 2) [287].

These diverse effects of CR on CSCs, which seem to vary depending on the tissue of origin, highlight the need for further research to fully understand the mechanisms and implications of CR on cancer stem cells.

## 4. Bioactive Compounds and Drugs in CR-Mediated Cancer Prevention

Bioactive compounds and drugs play a pivotal role in fueling the cancer-preventive effects of CR by mimicking its ability to modulate various biological pathways associated with cellular metabolism, oxidative stress, and inflammation. These compounds, including natural substances such as flavonoids, polyphenols, and other phytochemicals, emulate CR’s effect to optimize health benefits and reduce cancer risk. By influencing critical signaling pathways, such as those involved in cell growth, apoptosis, and autophagy, bioactive compounds not only replicate the physiological effects of CR but also offer potential therapeutic avenues for cancer prevention. Understanding the interplay between these compounds and CR mechanisms can provide valuable insights into developing comprehensive strategies for cancer management and prevention (Table 3) [153,292].

### 4.1. Quercetin: A Bioactive Compound in Cancer Prevention

Quercetin, a flavonoid widely found in fruits, vegetables, and grains, has emerged as a promising bioactive compound in cancer prevention. Its ability to modulate various cellular pathways positions it as a potential mimic of CR and fasting mimetics in the fight against cancer. Quercetin exhibits a diverse range of biological activities, including antioxidant, anti-inflammatory, and anti-cancer properties, making it a subject of interest in cancer research [294,306].

Mitigating oxidative stress through antioxidant activity

One of the primary mechanisms through which quercetin exerts its protective effects is by acting as a potent antioxidant. By scavenging ROS and enhancing the activity of endogenous antioxidant enzymes, quercetin mitigates oxidative stress, which is a well-established contributor to cancer development (Table 3). This action aligns with the oxidative stress reduction observed in CR. Thus, the complementary action of quercetin can amplify the protective effects on cellular integrity, thereby enhancing the resilience of normal cells while potentially targeting cancer cells more effectively [293].

Modulation of signaling pathways: Mechanistic Target of Rapamycin (mTOR)

Quercetin has been shown to inhibit the mTOR pathway, a central regulator of cell growth and metabolism. By downregulating mTOR activity, quercetin mimics the effects of CR, promoting autophagy and enhancing cellular repair mechanisms (Table 3).

Moreover, quercetin’s ability to induce apoptosis in cancer cells through the activation of pro-apoptotic proteins and inhibition of anti-apoptotic factors highlights its potential as a therapeutic agent. This action, in conjunction with CR and fasting, may contribute to the selective elimination of cancerous cells while sparing normal tissues [294].

Anti-inflammatory effects

Chronic inflammation is a recognized risk factor for cancer development, and quercetin’s anti-inflammatory properties provide another layer of protection. By inhibiting pro-inflammatory mediators such as cyclooxygenase (COX) and lipoxygenase (LOX), quercetin can reduce inflammation, thus lowering the risk of cancer initiation and progression (Table 3) [293]. The synergistic effects of quercetin with CR and fasting, which also promote an anti-inflammatory state, may further enhance the anti-cancer potential and be additionally helpful in cancer prevention [293,294,295].

### 4.2. Resveratrol: A Bioactive Compound in Cancer Prevention

Resveratrol is a natural polyphenolic compound predominantly found in grapes, berries, and certain nuts [307]. It has garnered significant attention for its potential health benefits, particularly in cancer prevention. By influencing key cellular pathways involved in metabolism and stress responses, resveratrol mimics the mechanisms of CR, offering a multifaceted approach to reducing cancer risk and promoting overall health [307,308].

Antioxidant properties

Resveratrol is a potent antioxidant that helps mitigate oxidative stress, a key contributor to cancer initiation and progression. By neutralizing ROS, resveratrol supports the protective effects of CR against cellular damage (Table 3) [296].

Modulation of signaling pathways: SIRT1

Resveratrol is known to activate SIRT1, a protein that plays a crucial role in extending lifespan and promoting cellular health. SIRT1 activation by resveratrol mimics some effects of CR, enhancing autophagy and improving metabolic health, thereby reducing cancer risk (Table 3) [297].

Modulation of inflammatory responses

Resveratrol exerts anti-inflammatory effects by inhibiting pro-inflammatory cytokines and pathways, such as NF-κB. This mechanism mirrors CR’s ability to promote an anti-inflammatory environment, which plays a vital role in cancer prevention (Table 3) [298].

### 4.3. Rapamycin: A Potential CR-Mimetic in Cancer Prevention

Rapamycin, a natural compound derived from Streptomyces hygroscopicus, has gained attention for its profound effects on cellular aging and cancer biology. As an inhibitor of the mTOR pathway, rapamycin mimics several cellular responses induced by CR, including autophagy enhancement and reduced cell proliferation. Its capacity to modulate inflammation and bolster stress resistance positions rapamycin as a promising CR-mimetic agent with potential applications in cancer prevention. By targeting pathways integral to both cellular metabolism and oncogenesis, rapamycin offers unique possibilities for reducing cancer risk [309].

Reduction in inflammatory responses through inhibition of Mechanistic Target of Rapamycin (mTOR) pathway

Rapamycin directly inhibits mTOR, a key pathway involved in cell growth, proliferation, and inflammation, which are crucial in preventing chronic inflammation—a known contributor to cancer development [299]. Similar to CR, rapamycin’s mTOR inhibition promotes autophagy and reduces cellular proliferation rates, thereby limiting the conditions that favor cancer growth (Table 3) [300].

Enhancement in cellular resilience to stress

Rapamycin has been shown to improve cellular stress resistance by enhancing autophagic processes, promoting cell repair, and preventing DNA damage [301]. This mechanism aligns with the protective effects of CR against cellular damage, adding a layer of resilience against cancer-promoting stressors (Table 3) [302].

### 4.4. Metformin: A Calorie Restriction Mimetic in Cancer Prevention

Metformin, a widely used antidiabetic drug, has shown potential as a CR mimetic due to its effects on cellular energy regulation and metabolism [310]. By activating AMPK and reducing insulin and IGF-1 signaling, metformin mirrors some of the cancer-protective effects of CR, promoting cellular resilience and reducing cancer-promoting pathways [311,312]. Its anti-inflammatory properties and metabolic modulation make metformin a promising candidate in strategies aimed at cancer prevention, offering insights into how CR-like mechanisms can be harnessed for therapeutic benefit [312].

AMP-activated protein kinase (AMPK) activation to regulate cellular energy balance and reduce inflammation

Metformin activates AMPK, a key energy-sensing enzyme. This activation mimics some effects of CR by shifting cellular metabolism toward energy efficiency and reducing excessive cellular proliferation, both of which are beneficial for cancer prevention (Table 3) [303,304].

Furthermore, AMPK-dependent pathways help decrease chronic inflammation, a known risk factor for cancer. This anti-inflammatory effect supports CR’s role in fostering a cellular environment less conducive to tumor formation (Table 3) [303].

Metformin-induced insulin reduction and Insulin-Like Growth Factor-1 (IGF-1) signaling to suppress cancer growth

Metformin lowers insulin levels and IGF-1 signaling, which can reduce the risk of cancer. Elevated insulin and IGF-1 are associated with increased cell growth and proliferation, while metformin’s influence on these pathways parallels CR’s ability to reduce cancer-promoting signals (Table 3) [305].

Metformin and clinical anti-cancer use

While metformin (N, N-dimethylbiguanide), a primary medication for managing type 2 diabetes mellitus, was initially introduced for clinical use in 1957, its importance as an anti-cancer drug was discovered nearly two decades later [313]. Serendipitously, while investigating the metabolic effects of metformin in diabetic patients, researchers found that metformin inhibits Complex 1 of the mitochondrial respiratory chain. This inhibition activates AMP-activated protein kinase (AMPK), recognized as a tumor suppressor, and plays a key role in regulating several downstream anabolic pathways that are crucial for tumor cell proliferation. This led to the development of drug repurposing programs that seek to utilize metformin for its role in suppressing tumor growth [314]. However, metformin alone in cancer treatment has the disadvantages of high dose concentrations and few targeted cancer types [313]. While regulatory hurdles, the lack of large-scale clinical trials, and inconsistent results in existing studies have played an important role in keeping metformin from being used popularly from a clinical perspective, clinical trials are still exploring the potential of repurposing metformin as a cancer therapeutic.

### 4.5. Challenges in Using Bioactive Compounds in Clinical Protocols

The integration of bioactive compounds such as quercetin, resveratrol, rapamycin, and metformin into clinical protocols for cancer prevention and therapy remains challenging despite promising preclinical evidence. Several barriers must be addressed to harness their potential effectively.

Contradictory findings in the literature

While bioactive compounds have been widely studied for their anti-cancer effects, inconsistencies in findings present a major challenge. For instance, resveratrol is often celebrated for its antioxidant properties [315] and tumor-suppressive effects [316], but certain studies have reported its pro-oxidant activity [317,318,319] under specific conditions, potentially exacerbating oxidative stress and promoting cancer progression [319,320]. Such contradictory outcomes complicate efforts to establish these compounds as reliable therapeutic agents.

Complexity of active components

Most bioactive compounds are derived from natural sources containing multiple synergistic components. The specific active components responsible for their observed biological effects are often not well defined. For example, quercetin is present in various plant-derived foods alongside other flavonoids, and their collective contribution to its anti-cancer effects remains unclear [321,322]. This lack of clarity hinders the development of standardized formulations with predictable efficacy.

Issues with bioavailability, stability, and efficacy

Bioavailability, stability, and efficacy are critical factors influencing the clinical applicability of bioactive compounds. Many bioactive compounds, such as quercetin [323,324,325] and resveratrol [326,327], have low solubility and rapid metabolism, resulting in limited systemic availability. Similarly, storage and transport conditions can compromise their stability, reducing their therapeutic potential. Strategies like nanoformulations and chemical modifications are being explored to overcome these limitations, but these approaches remain largely experimental [328,329].

Regulatory hurdles and clinical translation

Despite encouraging preclinical findings, the clinical use of bioactive compounds is hindered by stringent regulatory requirements and the lack of large-scale, robust clinical trials. Regulatory challenges are compounded by the variability in results from existing studies, which undermines confidence in their therapeutic value. A concerted effort to address these gaps through well-designed clinical trials and regulatory advocacy is essential for advancing the clinical application of bioactive compounds [330,331,332].

## 5. Concluding Remarks

While evidence-based recommendations, mainly coming from epidemiological studies, have already established that Mediterranean-like dietary patterns can help reduce cancer risk, DR interventions are emerging, from both preclinical and clinical research studies, as feasible and effective practices, which may also be taken into account as cancer-preventive approaches.

Although the precise molecular mechanisms remain incompletely understood, numerous basic and preclinical studies have demonstrated that various forms of DR can effectively reduce tumor incidence. Furthermore, earlier clinical studies indicate that reducing total calorie intake lowers cancer risk factors by enhancing cancer immunosurveillance, decreasing factors stimulating cell division, promoting autophagy, and improving metabolic health. These metabolic improvements include reductions in total cholesterol and triglyceride blood levels, diastolic blood pressure, glucose levels, IGF-1 levels, and ROS, along with increased insulin sensitivity. In addition, although the current available evidence mainly relies on preclinical studies, some types of DR have also been shown to positively influence gut microbiota composition, enriching the number of beneficial bacteria, which, in turn, may favorably influence host metabolism, gut barrier, and immune response, hence eventually delaying the onset of chronic diseases, such as cancer. To date, there is no evidence to suggest that one particular calorie-restricted regimen is more effective than another in preventing tumors. Indeed, they all act on the same cell proliferation pathways, making them potentially equally effective in controlling tumorigenesis. However, the molecular mechanisms activated by DRs, their effect on microbial species and, consequently, on human health remain to be elucidated, and further clinical and epidemiological research is needed to better understand their potential impact on cancer prevention. If this is proven, it will be appropriate to favor those that best maintain overall human health and are most readily accepted and integrated into daily life.

In addition, Calorie Restriction mimetics have been developed, some of which have already been approved as anti-cancer drugs and employed in specific clinical trials. Of course, compared to DR interventions, CRMs’ treatment outcomes are more predictable; moreover, the possibility of varying their administration protocols (as stand-alone and/or in combination with DRs), as well as dosages, represents a significant advantage for their clinical application. However, even though some CRMs are still under study and/or therapeutic validation, and novel CRMs might be discovered while dissecting DR’s mechanisms and effects on human health, they possess a large potential as a feasible and effective strategy against cancer and many other pathologies.

Considering the promising effects described in this review associated with CR, IF, and FMD-based interventions in vitro in preclinical studies and in the few available clinical studies, it is crucial to persist in exploring the effects of these and other DRs on human health. Specifically, more carefully designed clinical studies with larger sample sizes are warranted to fully ascertain the potential anti-tumor activity of these DRs, not only as stand-alone treatments but also in combination with CRMs and/or evidence-based recommended dietary approaches. In fact, it would be great to eventually create harmonized dietary and lifestyle guidelines that include these measures as effective cancer prevention procedures.

## Figures and Tables

**Table 2:** Effects of Calorie Restriction (CR) and fasting on stem cell biology.

Effect	Mechanism Description
Metabolic reprogramming	During periods of CR or fasting, a metabolic shift occurs that favors oxidative phosphorylation and fatty acid oxidation over glycolysis. This metabolic switch enhances stem cell quiescence, protecting them from environmental stress and cellular damage [278].
Modulation of stem cell function	Fasting promotes the self-renewal of hematopoietic stem cells, increasing their regenerative capacity. In addition, it has also been able to rejuvenate aged stem cells and restore their ability to produce new, functional cells. CR has been shown to preserve muscle stem cells, which is an important factor in muscle regeneration after injury [279,280].
Oxidative stress reduction	Both CR and fasting lower ROS levels by reducing mitochondrial activity and shifting the metabolic balance toward pathways that generate less oxidative damage, thus protecting stem cells from DNA damage and increasing their longevity and functional capacity [281].
Stem cell regeneration	During periods of nutrient deprivation, such as CR and fasting, autophagy is upregulated. This increased activity has been linked to improved stem cell regeneration in several tissues, including the brain, ameliorating cognitive function and neurogenesis, and muscle, which contributes to improved muscle repair [282,283].
Cancer stem cell activity	Fasting has been shown to selectively stress CSCs, making them more susceptible to chemotherapy, while at the same time protecting normal stem cells from the toxic effects of this treatment. This dual activity is mediated by changes in nutrient-sensing pathways, such as mTOR and AMPK [153,284]. However, while CR was found to suppress cancer stem cell populations in the MMTV-ERBB2 model [98,285], it increased a subset of cells similar to the CSC population in leukemia and enhanced the number and tumorigenic potential of intestinal stem cells [286,287].

**Table 3:** Effects of some bioactive compounds and drugs, alone and combined with Calorie Restriction (CR) and fasting, in cancer prevention.

Type of Bioactive Compound	Effect	Mechanism Description
Quercetin	Antioxidant activity	Quercetin reduces oxidative stress by scavenging ROS and increasing the activity of endogenous antioxidant enzymes [293]. The complementary action of quercetin may enhance the protective effects of CR on cellular integrity, thereby increasing the resilience of normal cells while potentially targeting cancer cells more effectively [293].
	Modulation of signaling pathways	Quercetin has been shown to inhibit the mTOR pathway. By downregulating mTOR activity, quercetin mimics CR’s effects by promoting autophagy and cellular repair [294]. This action, combined with CR and fasting, may help to selectively eliminate cancer cells while sparing normal tissue [294].
	Modulation of inflammatory responses	By inhibiting pro-inflammatory mediators, such as cyclooxygenase and lipoxygenase, quercetin may reduce inflammation, thereby decreasing the risk of cancer development and progression [293]. The synergistic effects of quercetin with CR and fasting may further enhance the anti-cancer potential and be additionally helpful in preventing cancer [293,294,295].
Resveratrol	Antioxidant activity	Resveratrol is a powerful antioxidant that helps reduce oxidative stress. Through the neutralization of ROS, it supports the protective effects of CR against cell damage [296].
	Modulation of signaling pathways	Resveratrol is known to activate SIRT1. Once activated, SIRT1 mimics some of the effects of CR by increasing autophagy and improving metabolic health, thereby reducing cancer risk [297].
	Modulation of inflammatory responses	Resveratrol exerts its anti-inflammatory effects by inhibiting pro-inflammatory cytokines and signaling pathways. This mechanism mirrors CR’s ability to promote an anti-inflammatory environment, which plays an important role in preventing cancer [298].
Rapamycin	Modulation of inflammatory responses	Rapamycin directly inhibits mTOR, thus promoting autophagy, reducing cellular proliferation rates, and preventing chronic inflammation, thereby limiting the conditions that favor cancer growth [299,300].
	Enhancement in cellular resilience	Rapamycin has been shown to improve the resistance of cells to stress by enhancing autophagic processes, promoting cell repair, and preventing DNA damage [301]. This mechanism parallels CR’s protective effects against cellular damage [302].
Metformin	Modulation of inflammatory responses	Metformin stimulates AMPK, activating specific pathways that can reduce chronic inflammation. This anti-inflammatory effect supports CR’s role in promoting a less tumorigenic cellular environment [303].
	Regulation of cellular energy balance	Metformin activates AMPK, thereby mimicking some of the effects of CR by shifting the cellular metabolism toward energy efficiency and reducing excessive cellular proliferation, both of which are beneficial in preventing cancer [303,304].
	Regulation of insulin levels and IGF-1 signaling	Metformin reduces insulin and IGF-1 levels, which boost cell growth and proliferation. Its effect on these pathways mirrors CR’s ability to reduce cancer-promoting signals [305].

## Data Availability

No new data were created or analyzed in this study. Data sharing is not applicable to this article.
